# Cognitive Care Planning for Persons Living With Dementia and Their Care Partners: Challenges, Needs, and Questions

**DOI:** 10.1093/geroni/igaf122.1140

**Published:** 2025-12-31

**Authors:** Katherine Britt, Rebecca Brown, Xiao Luo, Bin Huang

**Affiliations:** University of Iowa, Iowa City, Iowa, United States; University of Pennsylvania, Philadelphia, Pennsylvania, United States; Oklahoma State University, Stillwater, Oklahoma, United States; BrainCheck Inc, Austin, Texas, United States

## Abstract

Dementia care partners face high levels of stress, burden, and health risks related to the complex care demands of Alzheimer’s disease and Alzheimer’s disease related dementias (AD/ADRD). They also struggle with coordinating care, locating AD/ADRD resources and support, and managing AD/ADRD behavioral symptoms. Cognitive care planning (CCP) is a dementia care resource that provides care recommendations based on AD/ADRD needs assessments. CCP improves quality of life and overall care management, yet care partners lack ongoing support in implementing these CCPs at home. As dementia progresses and care challenges may escalate, ongoing support, resources, and innovative solutions for older adults living with AD/ADRD and care partners are needed. We aim to develop a tailored AI-based chatbot to support dyads by first exploring potential challenges, needs, and questions dyads encounter when implementing their CCPs in daily life. We recruited older adults living at home with mild cognitive impairment or mild AD/ADRD and their care partners (N = 20 dyads) and dementia care experts (N = 10) from U.S. medical centers and clinics in the Pacific Northwest and the University of Pennsylvania. We conducted semi-structured interviews and will use content analysis to analyze data and report qualitative categories and subcategories denoting common and individualized questions gathered from participants. Findings will be used to inform development of an AI chatbot designed to provide continuous and real-time care support for dyads to improve quality of life, decrease care partner burden, and improve care management.

